# 

**Published:** 1889-09-14

**Authors:** 


No. 155, Vol. VI.—September 14th, 1889.
Registered at the General Post Office as a Newspaper.]

Monthly Parts, 6d.; post free 7½d.

Ditto per Annum, 7s. 6d., post free.

### THE HOSPITAL

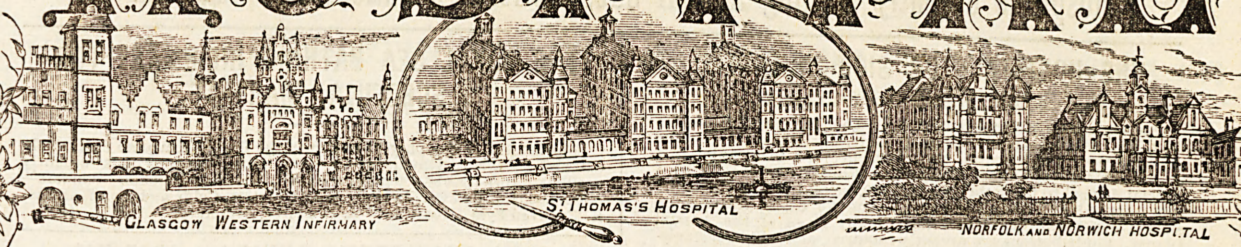

A WEEKLY INSTITUTIONAL JOURNAL OF

Science, Medicine, Nursing & Philanthropy.

### SPECIAL STUDENTS' NUMBER.

